# Life cycle impact assessment of metal production industries in Australia

**DOI:** 10.1038/s41598-021-89567-9

**Published:** 2021-05-12

**Authors:** Vladimir Strezov, Xiaoteng Zhou, Tim J. Evans

**Affiliations:** Department of Earth and Environmental Sciences, Faculty of Science and Engineering, Macquarie University, Sydney, NSW 2109 Australia

**Keywords:** Environmental impact, Metals and alloys

## Abstract

Metal production industries are associated with positive economic benefits, however their activities are significantly resource and energy intensive, contributing to emission of pollutants and greenhouse gases to the environment. The balance between the economic inputs and environmental footprint of the metal production industries determines their contribution to sustainability. This work provides environmental impact assessment of the production of aluminium, copper, gold, iron and steel, lead, nickel and zinc, and considers their contribution to the economy. The emissions of selected representative industries in Australia were sourced from public national emission inventories and used as input parameters in the openLCA software. ReCiPe midpoint and endpoint hierarchist impact assessment methods were used to investigate the environmental impacts of the selected industries. The results indicate that lead, followed by aluminium and nickel production had the largest environmental impacts. The work further revealed the specific emissions for better control for each industry taking into consideration their relative environmental and economic impacts. For instance, adoption of renewable energy sources would significantly decrease the greenhouse gas emissions and the associated environmental impacts of the copper, zinc, gold, and iron and steel production industries. Improvement of sustainability of the production of lead would require further control of trace metal emissions, while for aluminium and nickel production, improved control of emissions of particles and the acidic gases SO_2_ and NO_x_.

## Introduction

Production of metals has significant input to global economies with their wide and diverse applications in everyday life. However, production and refining of metals are some of the most energy intensive and highly environmentally challenging industrial activities^1^, emitting greenhouse gases, particles, trace metals, acidic gases and organic pollutants to the environment^2^. The balance between the economic benefits and environmental impacts is one of the ways by which industrial sustainability can be assessed.

There have been different attempts to investigate the parameters of importance to define sustainability of industrial operations. It is generally accepted that direct emissions of greenhouse gases and pollutants are some of the most important parameters for defining sustainability of industrial processes^3^. Conservation of natural resources and waste reduction^4^, increasing the product value^5^, including social dimension of sustainability^6^ have also been highlighted as important parameters for industrial sustainability. Norgate and Haque^7^ applied life cycle assessment (LCA) as a tool to determine the environmental impacts of a range of metal production and confirmed its importance as a method for wider impact assessment of the industries.

The most widely used LCA software tools are SimaPro, GaBi, Umberto® and openLCA^8^. However, there is a wide range of developed life cycle impact assessment (LCIA) methods, which can be selected depending on the objectives of the study. There is currently no standard and uniformly accepted impact assessment method, although Hauschild et al.^9^ identified ReCiPe and USETox as the best midpoint, and midpoint to endpoint characterisation methods.

Previous studies applied ReCiPe or USETox impact assessment methods to assess environmental impacts during metal processing in individual countries, such as Australia^10^, China^11^, Norway^12^ and Poland^13^, as well as at a global scale^14^. The results showed that metal production generates a range of gaseous and particulate matter (PM) emissions during metal and mineral processing (i.e. crushing, grinding, sizing, drying and calcining)^15^. As a result, the related environmental implications, such as global warming and pollution, have become a global issue, posing a significant risk to both human and ecosystem health^16^.

Mineral industries also provide significant economic benefits to the society, as is the case with gold^17^. Although 3R (Reduce, Reuse and Recycle) measures were proposed to improve sustainability^18^, developing a standard method to achieve balance between the environmental impacts and economic development is still a challenge. During the sustainability assessment process, the use of datasets is the most critical step.

Currently, the input parameters of emission data in the LCIA methods are typically based on direct measurements^19^ or they rely on commercial databases^20^. There is no LCIA study of mineral processing industries that consider national emission inventories, which are publicly available emission databases. The national pollutant inventories have been shown to provide a source of data that can be used to estimate environmental impacts of power generation technologies^21^. This work presents for the first time LCIA study of environmental and economic impact assessment of Australian metal production industries using the emission data of the Australian national pollutant inventory to determine the relative performance of different industries, as well as reveal the areas which require further improvement and control by the industrial processes in order to reduce their emissions and environmental impacts while still contributing to the national economic development. The study focuses on metal production in Australia as it is one of the leading producers of minerals in the world^22^. The scope of the research is to identify the specific environmental impacts from emission of pollutants of each of the metal production industries in Australia relative to their economic contribution that may require further control to achieve improved sustainability.

## Methods

**Figure 1:**
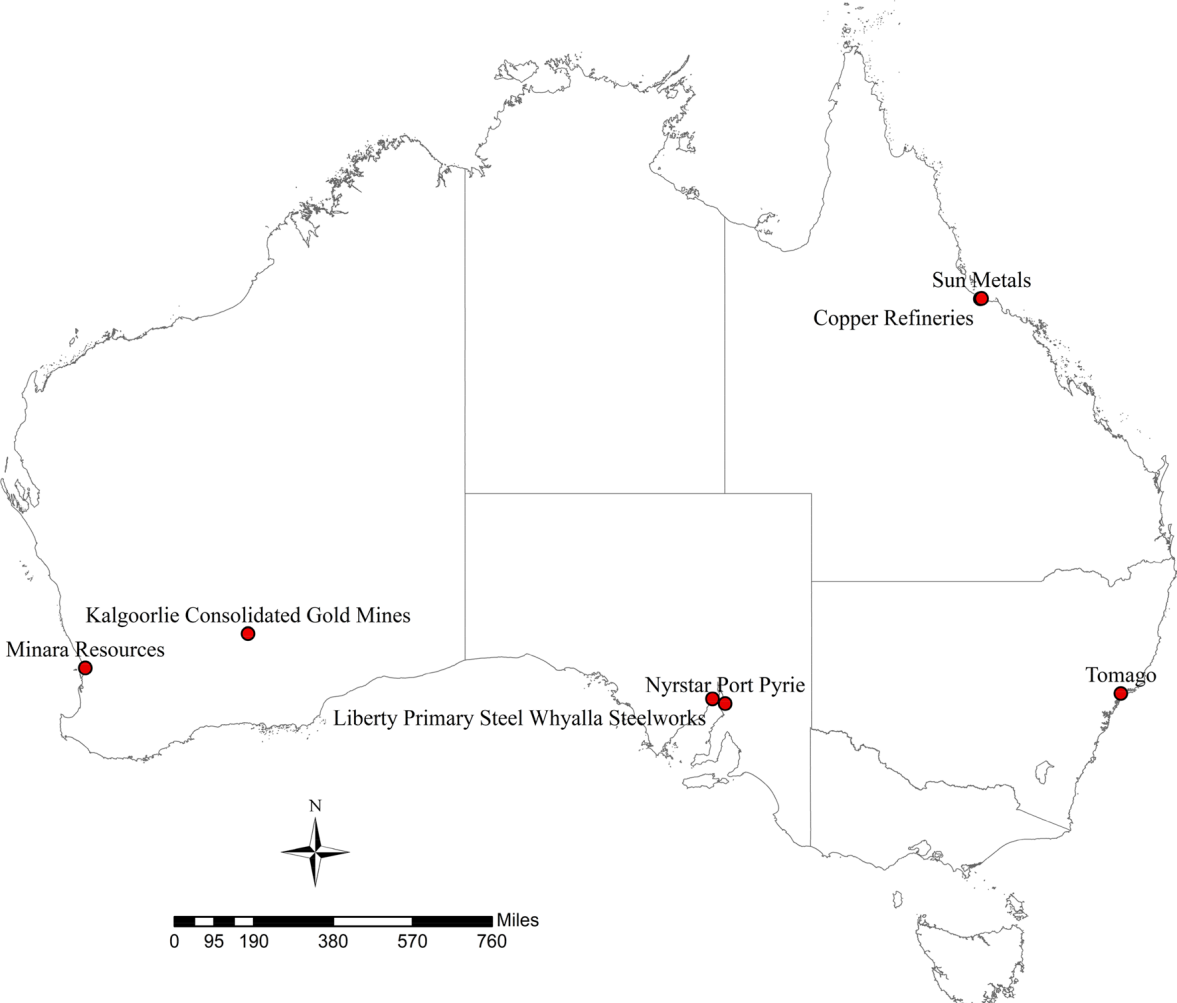
Map of the locations for the seven companies investigated in this study. The map was constructed with ArcGIS 10.6 software (https://www.esri.com).

Seven companies based in Australia producing aluminium, copper, gold, iron and steel, lead, nickel, and zinc, were selected for this study to determine their environmental impacts. The companies with their annual production for the period of July 2017 to June 2018 are presented in Table 1 with Fig. 1 showing the map of their locations in Australia. Iron and steel production with 1.2 Mt had the largest amount of produced metals, followed by aluminium at 585 kt/year, while gold production with less than 20 t/year had the lowest amount of metal produced, followed by nickel at 39.7 kt/year.

**Table 1:** Selected companies based in Australia which produce metals.

Metal type	Company	Annual production (t)
Iron and steel (Fe)	Liberty primary steel Whyalla steelworks	1,200,000
Aluminium (Al)	Tomago	585,000
Copper (Cu)	Copper refineries	300,000
Nickel (Ni)	Minara resources	39,717
Lead (Pb)	Nyrstar port pyrie	170,000
Zinc (Zn)	Sun metals	250,000
Gold (Au)	Kalgoorlie consolidated gold mines	19.8

The direct emissions of pollutants to air, water and soil from each of the selected companies were sourced from the National Pollutant Inventory (NPI)^23^ for the period of July 2017 to June 2018. Considering Minara Resources produced small amounts of cobalt in their process, the emissions of this industry for nickel production were calculated by multiplying the total emissions with 0.9245, which was the fraction of nickel produced per tonne of total metal by this company. The greenhouse gas emissions for each company were sourced from the corporate emissions and energy data, and the safeguard baseline tables published by the Clean Energy Regulator^24^ for the same period of time.

The goal of the study was to investigate the environmental impacts of the metal production industries in Australia. The system boundaries in the study were the direct point source emissions to air, water and soil, as defined by the emission reporting requirements for the National Pollutant Inventory. Two functional units were used to express the impacts based on per tonne of produced metal and per US$1000 of revenue from production of the metals. For the latter, in case of iron and steel, the market cost of hammered round iron (HRB) price on 25 June 2018 was used^25^ while for the prices of all other metals the InvestmentMine^26^ source was used.

**Figure 2:**
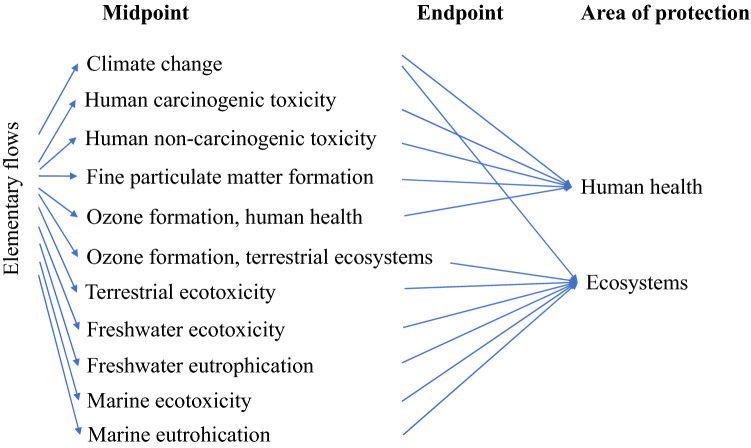
Graphic presentation of the link between the used midpoint and endpoint impact assessment categories ( modified from Huijbregts et al.^28^).

openLCA 1.10.3 software^27^ (https://www.openlca.org/) and ReCiPe 2016 Midpoint and Endpoint hierarchist impact assessment methods^28^ were used to determine the environmental impacts of the selected metal production industries in Australia. The impact assessments considered in this work are graphically shown in Fig. 2. The endpoint impact categories were divided into human health impacts and impacts on ecosystems, expressed in disability adjusted life years (DALY) and loss of species during a year (species.yr), respectively.

The emission data from the NPI database were used as input source in the openLCA software. Low population density and unspecified type of land and water were selected as elementary flow categories. Once the data was included in the software, ReCiPe 2016 Midpoint and Endpoint hierarchist methods were executed to conduct the impact assessment for the selected categories shown in Fig. 2.

## Results

Table 2 shows the atmospheric emissions of only the criteria air pollutants, priority metals and priority air toxics based on per tonne of produced metal. The health and environmental impacts of each of the listed substances have been summarised in supplementary Table S1, in accordance to NPI^29^. Due to the highly energy intensive process to produce gold, according to Table 2, the overall emissions of gold production were found to be significantly higher than the production of base metals. This was consistent with previous local studies which concluded that gold processing generated higher environmental waste compared to other metal industries^30^. Previous studies also demonstrated that gold processing was a large contributor to local atmospheric pollution, including emission of PM^31^, gaseous pollutants, organic and metal contaminants^32^. This study also revealed that from the selected industries, production of gold was the only industry emitting formaldehyde, which is used as a reducing agent.

**Table 2:** Atmospheric emissions of criteria and priority atmospheric pollutants expressed on per tonne of metal produced.

	Fe	Al	Cu	Ni	Pb	Zn	Au
**Criteria air pollutants (kg/t)**							
NO_x_	2.38	0.606	4.0E−02	14.2	6.63	7.5E−02	9.4E + 04
SO_2_	0.907	19.0	4.6E−05	28.3	366	2.42	64.5
CO	46.6	81.0	0.344	6.27	194	4.4E−02	3.6E + 04
PM_10_	2.44	0.152	2.7E−03	366	1.08	7.0E−02	1.5E + 05
PM_2.5_	0.329	9.0E−02	2.7E−03	0.99	0.166	4.6E−03	5640
VOCs	1.23	1.1E−02	2.8E−03	0.937	0.102	7.4E−03	5448
**Priority metals (g/t)**							
As	3.0E−02	4.1E−02	5.1E−03	5.11	19.3	0.549	3.6E + 04
Cd	2.8E−03	0.111	4.8E−04	0.669	10.7	0.294	62.8
Cr(VI)	2.6E−03	1.4E−02	1.9E−04	1.5E−02	3.5E−02	3.2E−05	0
Cu	0.416	7.9E−02	0.279	27.9	3.67	8.5E−02	2.8E + 04
Pb	7.1E−02	0.130	7.1E−03	14.5	341	21.3	2884
Hg	1.3E−03	5.8E−03	2.7E−04	0.108	5.17	4.1E−02	1.3E + 04
Ni	6.7E−02	0.318	4.0E−03	3199	0.105	6.4E−03	1.4E + 04
Se	0	0	0	0	16.6	0	384
**Priority air toxics (g/t)**							
Benzene	19.7	0	0	5.0E−02	0	0	1.4E + 04
Formaldehyde	0	0	0	0	0	0	1.3E + 06
Dioxins and furans (TEQ)	1.4E−07	6.8E−09	2.2E−09	0	3.9E−06	1.4E−09	8.2E−06
PAH (B[a]Peq)	0.383	3.6E−02	4.1E−04	0.2	0.523	2.5E−03	1034
Toluene	2.84	0	0	0.586	0	0	6113
Xylenes	0.574	0	0	0.437	0	0	4551

The emission of criteria pollutants for production of base metals were the highest for nickel production with emissions of NO_x_, SO_2_ and both PM_10_ and PM_2.5_. The high atmospheric emissions generated during nickel production was also found by a Finnish study^33^. Iron and steel emitted the highest CO and volatile organic compounds (VOC) as well as the highest amounts of benzene, toluene and xylenes, which have been confirmed by Tsai et al.^34^ and Chang et al.^35^. Lead production emitted the highest amount of all metals, except for Cu and Ni, which were emitted the highest by the nickel production industry. Lead production also emitted the highest amounts of dioxins and furans, and polycyclic aromatic hydrocarbons (PAHs).

Table 3 shows the emissions of the criteria pollutants expressed on per US$1000 of market price of each metal. This is specifically important when comparing emissions and impacts of industries producing different value products. In case of gold production, the economic based emission reporting determines more accurately the overall impacts when compared to emissions from base metal production. Table 3 shows that, based on economic value, lead production emitted the highest amounts of pollutants, including NO_x_, SO_2_, CO, As, Cd, Cr(VI), Co, Pb, Hg, Se and dioxins. Iron and steel production emitted the highest amounts of PM_2.5_, VOCs, benzene, PAH, toluene and xylene, while nickel production emitted the highest amounts of PM_10_ and Ni.

**Table 3:** Atmospheric emission of criteria and priority atmospheric pollutants expressed on per US$ of metal market value.

	Fe	Al	Cu	Ni	Pb	Zn	Au
**Criteria air pollutants (kg/US$1000)**							
NO_x_	2.62	0.344	6.9E−03	0.845	3.04	3.0E−02	1.94
SO_2_	1.00	10.8	7.9E−06	1.69	168	0.955	1.3E−03
CO	51.4	45.9	5.9E−02	0.374	88.7	1.7E−02	0.738
PM_10_	2.69	8.6E−02	4.7E−04	21.8	0.493	2.8E−02	3.18
PM_2.5_	0.363	5.1E−02	4.7E−04	5.9E−02	7.6E−02	1.8E−03	0.116
VOCs	1.36	6.4E−03	4.8E−04	5.6E−02	4.7E−02	2.9E−03	0.112
**Priority metals (g/US$1000)**							
As	3.4E−02	2.3E−02	8.7E−04	0.305	8.84	0.216	0.742
Cd	3.1E−03	6.3E−02	8.2E−05	4.0E−02	4.90	0.116	1.3E−03
Cr(VI)	2.9E−03	7.8E−03	3.2E−05	8.9E−04	1.6E−02	1.3E−05	0
Cu	0.458	4.5E−02	4.8E−02	1.66	1.68	3.4E−02	0.574
Pb	7.8E−02	7.4E−02	1.2E−03	0.868	156	8.40	6.0E−02
Hg	1.4E−03	3.3E−03	4.6E−05	6.4E−03	2.37	1.6E−02	0.272
Ni	7.3E−02	0.180	6.9E−04	191	4.8E−02	2.5E−03	0.285
Se	0	0	0	0	7.62	0	7.9E−03
**Priority air toxics (g/US$1000)**							
Benzene	21.7	0	0	3.0E−03	0	0	0.286
Formaldehyde	0	0	0	0	0	0	26.8
Dioxins and furans (TEQ)	1.6E−07	3.9E−09	3.8E−10	0	1.8E−06	5.5E−10	1.7E−10
PAH (B[a]Peq)	0.422	2.0E−02	6.9E−05	1.2E−02	0.240	1.0E−03	2.1E−02
Toluene	3.13	0	0	3.5E−02	0	0	0.126
Xylenes	0.633	0	0	2.6E−02	0	0	9.4E−02

Table 4 compares the environmental impacts of the selected metal production industries expressed on per tonne of produced metal. Results showed that gold, nickel and lead production in Australia have higher environmental risk impacts than the other metals. Productions of these three metals generate large quantities of waste during processing, particularly the gold production which is estimated to release 99% of the extracted ore as waste^36^. In addition, gold, nickel and lead processing requires large amounts of chemicals for extraction and refining^15,37,38^, generating environmental burdens with regards to different environmental profiles at the midpoint and endpoint categories in the environmental impact assessment.

**Table 4:** Environmental impact assessment of metal producing industries expressed on per tonne of produced metal.

	Fe	Al	Cu	Ni	Pb	Zn	Au
**Midpoint**							
Human carcinogenic toxicity	0.153	0.552	4.3E−02	1610	23.1	0.872	3.2E + 04
Human non-carcinogenic toxicity	33.4	40.6	8.91	2482	18,363	1499	6.0E + 06
Ozone formation, Human health	2.38	0.606	4.0E−02	14.2	6.63	7.5E−02	9.4E + 04
Fine particulate matter formation	0.854	5.67	2.0E−02	12.3	107	0.715	1.6E + 04
Terrestrial ecotoxicity	952	561	493	945,601	94,976	14,507	8.8E + 07
Ozone formation, Terrestrial ecosystems	2.38	0.606	4.0E−02	14.2	6.63	7.5E−02	9.5E + 04
Terrestrial acidification	1.76	19.2	6.0E−02	46.7	368	2.46	3.4E + 04
Freshwater ecotoxicity	0.390	0.223	7.2E−02	6.23	39.4	0.415	933
Freshwater eutrophication	9.6E−05	0	0	0	6.0E−03	0	0
Marine ecotoxicity	1.03	0.661	0.290	522	105	11.1	3.5E + 04
Marine eutrophication	4.4E−02	0	0	0	0	0	0
Global warming	909	1988	2126	12,234	1981	2826	2.5E + 07
**Endpoint**							
Global warming, human health	8.4E−04	1.8E−03	2.0E−03	1.2E−02	1.8E−03	2.6E−03	23.0
Human carcinogenic toxicity	5.1E−07	1.8E−06	1.4E−07	5.8E−03	7.7E−05	2.9E−06	0.105
Human non-carcinogenic toxicity	7.6E−06	9.2E−06	2.0E−06	6.1E−04	4.2E−03	3.4E−04	1.36
Fine particulate matter formation, human health	5.4E−04	3.6E−03	1.3E−05	7.8E−03	6.7E−02	4.5E−04	10.1
Ozone formation, human health	2.2E−06	5.5E−07	3.6E−08	1.4E−05	6.0E−06	6.9E−08	0.086
Global warming, terrestrial ecosystems	2.5E−06	5.6E−06	6.0E−06	3.7E−05	5.5E−06	7.9E−06	0.069
Terrestrial acidification	3.7E−07	4.1E−06	1.3E−08	1.1E−05	7.8E−05	5.2E−07	7.2E−03
Terrestrial ecotoxicity	1.1E−08	6.4E−09	5.6E−09	1.2E−05	1.1E−06	1.7E−07	1.0E−03
Global warming, freshwater ecosystems	7.0E−11	1.5E−10	1.6E-10	1.0E−09	1.5E−10	2.2E−10	1.9E−06
Freshwater ecotoxicity	2.7E−10	1.5E−10	5.0E−11	4.7E−09	2.7E−08	2.9E−10	6.5E−07
Freshwater eutrophication	6.4E−11	0	0	0	4.0E−09	0	0
Marine ecotoxicity	1.1E−10	6.9E−11	3.0E−11	5.9E−08	1.1E−08	1.2E−09	3.7E−06
Marine eutrophication	7.4E−11	0	0	0	0	0	0
Human health total (DALY)	1.39E−03	5.41E−03	1.99E−03	2.65E−02	7.32E−02	3.42E−03	34.6
Ecosystems total (species year)	2.93E−06	9.65E−06	5.97E−06	5.96E−05	8.47E−05	8.60E−06	0.078

Production of gold had the highest impacts when considering the amount of metal produced and reflecting on the pollutant emissions released to extract gold. Previous LCIA studies also showed that, based on a per kilogram, the gold production presented greater environmental burden than the base metal processing^38^. Specifically, the greenhouse gas emissions released during gold processing is a major challenge in both Australia^38^ and internationally^39^. According to Mudd^30^, gold mining activities in Australia generated an average of 10.7–16.7 t CO_2_ per kilogram of produced Au during 1991 to 2005, while Norgate and Haque^38^ estimated emissions of 18 t CO_2_ eq/kg for production of gold. This study revealed that gold processing in Australia contributed to slightly higher global warming with an estimated 25 t CO_2_ eq per kg of produced gold (Table 4).

The atmospheric emissions generated during gold production also posed a significant risk to surface water health via deposition^40^. This study found that emission of zinc and copper during gold production had the highest impact contribution to freshwater and marine ecotoxicity. Previous local and international LCIA studies also confirmed the freshwater and marine ecotoxicity caused by gold processing activities^30^.

In addition to the environmental impacts, atmospheric emissions generated during gold production was also found to pose a risk to human health. The largest contributor to human toxicity from gold production, in both carcinogenic and non-carcinogenic category, was release of arsenic followed by atmospheric nickel and zinc emissions. The emission of NO_x_ was the highest contributor to fine particulate matter formation, ozone formation impacts on human health and ecosystems, and terrestrial acidification. Climate change impacts on human health and ecosystem quality were the other two major impact categories from gold production.

From the production of selected base metals in this study, production of lead had overall the highest impacts on per tonne of metal production basis in human non-carcinogenic toxicity, due to impacts from emissions of metals, such as lead, zinc and arsenic. Lead production also had high fine particulate matter formation as a result of release of PM_2.5_ formation precursors SO_2_ and NO_x_^15^, which also impacted the terrestrial acidification.

Nickel production was the following industry with high environmental impacts due to its human carcinogenic toxicity, ozone formation impacts on human health and terrestrial ecosystems, terrestrial ecotoxicity, marine ecotoxicity and marine eutrophication. The high environmental impacts of nickel production were previously related to high volumes of rocks mined in complex lateritic ores in Australia and large quantities of acid solvents required for extraction^37^.

Iron and steel production had the lowest overall impacts based on per tonne of produced metal, which was also found in another LCIA study where iron and steel production had lower environmental impacts than the other metal production industries^41^.

Table 5 shows the percentage distribution of the endpoint impacts based on the reported emissions to air, water and soil by each of the considered metal production companies. It is evident that the emissions to air have by far the most significant impacts, while emissions to water, followed by emissions to soil were comparatively significantly smaller. The largest human health impacts from emissions to wastewater were reported by the lead production industry with emissions of lead and zinc as the major contributors, followed by iron and steel production due to emissions of zinc in the wastewater stream.

**Table 5:** Percentage contribution of emissions to air, water and soil on human health and ecosystem endpoint impacts.

	Impact type	Air (%)	Water	Soil
Fe	Human health	99.755	0.245%	0
	Ecosystems	99.986	0.014%	0
Al	Human health	99.965	0.035%	0
	Ecosystems	99.998	0.002%	0
Cu	Human health	99.904	0.093%	0.003%
	Ecosystems	99.999033	0.000964%	0.000003%
Ni	Human health	99.998	0	0.002%
	Ecosystems	99.999998	0	0.000002%
Pb	Human health	99.467	0.533%	0
	Ecosystems	99.958271	0.041727%	0.000002%
Zn	Human health	99.999	0	0.001%
	Ecosystems	99.99998	0	0.00002%
Au	Human health	99.985	0	0.015%
	Ecosystems	99.9996	0	0.0004%

When considering the economic value of the metal production, lead production followed by aluminium production had the highest impacts, as shown in Table 6. Lead production had the highest impacts across all categories, except for human carcinogenic toxicity and terrestrial ecotoxicity in which it ranked the second, and global warming impacts in which lead production ranked fourth. Aluminium production ranked the second most impactful metal producing industry because of global warming impacts on human health and ecosystems, in which it ranked first, and fine particulate formation, in which it ranked second. The major contributor to fine particulate formation during aluminium production was assigned to SO_2_ emissions, in which aluminium production ranked the second after lead production, as shown in Table 3. Gold production, due to high value of the end product, showed significant reduction in overall impacts when economic parameters are taken into consideration. Production of nickel, zinc, and iron and steel had similar levels of environmental impacts with total endpoint impacts ranging between 1.3E−3 and 1.6E−3 DALY/US$1000 and 3.2E−6 to 3.6E−6 species.yr/US$1000. The results also showed aluminium and zinc have the highest greenhouse gas emissions of over 1100 kgCO_2_-eq/US$1000, while copper production followed by gold production are presented with the lowest greenhouse gas emissions based on the economic parameters.

**Table 6:** Environmental impact assessment of metal producing industries expressed on per US$1000 of metal market value.

	Fe	Al	Cu	Ni	Pb	Zn	Au
**Midpoint**							
Human carcinogenic toxicity	0.169	0.313	7.3E−03	96.1	10.6	0.344	0.654
Human non-carcinogenic toxicity	36.8	23.0	1.53	148	8413	591	123
Ozone formation, Human health	2.62	0.344	6.9E−03	0.845	3.04	3.0E−02	1.95
Fine particulate matter formation	0.942	3.21	3.4E−03	0.737	49.0	0.282	0.331
Terrestrial ecotoxicity	1050	318	84.5	56,436	43,516	5722	1823
Ozone formation, Terrestrial ecosystems	2.62	0.344	6.9E−03	0.845	3.04	3.0E−02	1.95
Terrestrial acidification	1.94	10.9	1.0E−02	2.79	169	0.970	0.701
Freshwater ecotoxicity	0.430	0.127	1.2E−02	0.372	18.0	0.164	1.9E−02
Freshwater eutrophication	1.1E−04	0	0	0	2.7E−03	0	0
Marine ecotoxicity	1.14	0.375	5.0E−02	31.1	48.1	4.36	0.720
Marine eutrophication	4.8E−02	0	0	0	0	0	0
Global warming	1002	1127	364	730	908	1115	512
**Endpoint**							
Global warming, human health	9.3E−04	1.0E−03	3.4E−04	7.3E−04	8.4E−04	1.0E−03	4.8E−04
Human carcinogenic toxicity	5.6E−07	1.0E−06	2.4E−08	3.5E−04	3.5E−05	1.1E−06	2.2E−06
Human non-carcinogenic toxicity	8.4E−06	5.2E−06	3.5E−07	3.7E−05	1.9E−03	1.3E−04	2.8E−05
Fine particulate matter formation, human health	5.9E−04	2.0E−03	2.2E−06	4.6E−04	3.1E−02	1.8E−04	2.1E−04
Ozone formation, human health	2.4E−06	3.1E−07	6.2E−09	8.3E−07	2.8E−06	2.7E−08	1.8E−06
Global warming, terrestrial ecosystems	2.8E−06	3.2E−06	1.0E−06	2.2E−06	2.5E−06	3.1E−06	1.4E−06
Terrestrial acidification	4.1E−07	2.3E−06	2.2E−09	6.4E−07	3.6E−05	2.1E−07	1.5E−07
Terrestrial ecotoxicity	1.2E−08	3.6E−09	9.7E−10	7.0E−07	5.0E−07	6.5E−08	2.1E−08
Global warming, freshwater ecosystems	7.7E−11	8.6E−11	2.8E−11	6.0E−11	6.9E−11	8.5E−11	3.9E−11
Freshwater ecotoxicity	3.0E−10	8.8E−11	8.6E−12	2.8E−10	1.2E−08	1.1E−10	1.3E−11
Freshwater eutrophication	7.1E−11	0	0	0	1.8E−09	0	0
Marine ecotoxicity	1.2E−10	3.9E−11	5.2E−12	3.5E−09	5.0E−09	4.6E−10	7.6E−11
Marine eutrophication	8.2E−11	0	0	0	0	0	0
Human health (total)	1.5E−03	3.1E−03	3.4E−04	1.6E−03	3.4E−02	1.3E−03	7.2E−04
Ecosystem quality (total)	3.2E−06	5.5E−06	1.0E−06	3.6E−06	3.9E−05	3.4E−06	1.6E−06

Table 7 shows comparison of the overall endpoint impacts of metal producing industries expressed per US$1000 and excluding the climate change impacts. This could be hypothetically achieved when all energy requirements are supplied from renewable energy sources. Adoption of renewable energy sources could significantly reduce the greenhouse gas emissions and improve the overall environmental impacts for all metal production industries, except for lead, for which the environmental impact contributions rely on direct emissions of particles, acidic gases and trace metals. The highest environmental impact reduction with adoption of renewable energy sources can be achieved by the copper, zinc, gold, and iron and steel production industries. Human health impacts from particulate matter formation is the largest impactful category among all industries as a result of direct particulate matter (PM) emissions and emission of PM formation precursors, such as NO_x_ and SO_2_. This is specifically the case for lead and aluminium production. The environmental performance of lead production can be improved by targeting better control of trace metal emissions, while in case of aluminium production the environmental performance can be improved with more efficient control of PM, SO_2_ and NO_x_.

**Table 7:** Total endpoint environmental impact assessment of metal producing industries expressed in points/US$1000 and excluding climate change impacts.

	Fe	Al	Cu	Ni	Pb	Zn	Au
Human health total (DALY)	6.0E−04	2.0E−03	2.5E−06	8.5E−04	3.3E−02	3.1E−04	2.4E−04
Ecosystem quality total (species year)	4.2E−07	2.3E−06	3.2E−09	1.3E−06	3.6E−05	2.7E−07	1.7E−07

Improved control of atmospheric emissions of trace metals, particles and acidic gases can be achieved through two main strategies, as stated by Karell^42^, which are pollution prevention an end-of-pipe technologies for removal and treatment of pollutants. The pollution prevention strategy is more cost effective but has limits, while the end-of-pipe technologies require capital investment and regular maintenance. Improved control of trace metals and PM with end-of pipe technologies can typically be achieved with cyclones as preconditioning units followed by fabric filters or electrostatic precipitators designed specific to the operating conditions of the plant. Acidic gases can be controlled using scrubbers for SO_2_ control and catalytic or non-catalytic reduction systems for NO_x_ control. Due to the diverse plant processes, several different pollution control technologies and approaches may be required to effectively reduce all pollutants^42^.

## Conclusion

This work demonstrates application of national pollutant inventories of industrial emissions of greenhouse gases and pollutants to air, water and soil for assessment of their environmental impacts. National pollutant inventories are free online government managed databases which can provide source of information for assessment of the specific emissions of importance for further control in order to achieve continuous environmental improvement. In this work seven metal producing companies operating in Australia as representatives of the iron and steel production industry, aluminium, copper, nickel, lead, zinc and gold production were selected to assess and compare their overall environmental performance. The pollutant emission data from the Australian National Pollutant Inventory and Clean Energy Regulator in Australia were used for environmental impact assessment using the openLCA software. ReCiPe midpoint and endpoint hierarchist methods were used to assess the overall impacts based on production per tonne of metal and per US1000 value of the produced metal. Results revealed that presenting emissions based on economic value gives better overview for comparison between the different range of industries. Production of lead, followed by aluminium and nickel were the most impactful metal producing industries where emissions of trace metals, particulate matter and acidic gases SO_2_ and NO_x_ had significant respective environmental impacts, which would require better control in order to improve sustainability of these industries. The study found that energy intensity of almost all metal production processes has considerable overall environmental impacts and adoption of renewable energy sources would improve the environmental performance of the copper, zinc, gold, and iron and steel production industries.

## Supplementary Information

Supplementary Information.
