# Supplementary material for: Life cycle impact assessment of metal production industries in Australia
Source: Sci Rep. 2021 May 12;11:10116. doi: 10.1038/s41598-021-89567-9 (PMC8115058; doi:10.1038/s41598-021-89567-9)
Supplement: Supplementary file 1 — Supplementary Information. [file 41598_2021_89567_MOESM1_ESM.docx]

Table S1. Health and environmental impacts of selected criteria and priority atmospheric pollutants (NPI, 2021).

| Substance | Health effects | Environmental effects |
| --- | --- | --- |
| SO_2_ | Irritation of eyes, nose and throat, choking and coughing, burns, effect on vision, inflammation of the respiratory tract, wheezing and lung damage | Harmful to plants and trees, reduction of crop productivity, causes acid rain, affects land and water ecosystems |
| NO_2_ | Irritation of eyes, nose, throat and lungs, causes coughing, shortness of breath, tiredness and nausea, burning, build-up of fluid in lungs | Damage to leaves of plants, damage to crop production, causes photochemical smog and acid rain |
| CO | Low levels cause headache, dizziness, light-headedness and fatigue, while high doses cause sleepiness, loss of consciousness and death | Oxidises to CO_2_,which is a greenhouse gas, can affect health of birds and animals |
| PM | Allergic effects, bacterial and fungal infection, irritation to mucus membranes, aggravation of asthma and premature mortality | Animals are affected in the same way as humans. PM contribute to reduced visibility |
| VOCs | Irritation of eyes, nose and throat; headaches; loss of coordination; nausea; damage to the liver, kidney and central nervous system; some VOCs are carcinogenic | VOCs contribute to photochemical smog formation, animals are affected in the same way as humans |
| As | Digestive tract pain, nausea, vomiting, decreased production of blood cells, damage to blood vessels, abnormal heart rhythms and death | high toxicity to aquatic life, birds and animals |
| Cd | Causes kidney and prostate cancer, teratogen, causes lung damage | Persistent in the environment causing the same effects in animals as in humans |
| Cr(VI) | Irritation of nose, throat, lungs, stomach and intestines, causes asthma, stomach upsets, kidney and liver damage, adverse effects on the respiratory and the immune systems and can cause cancer | Acute toxicity on plants, birds or land animals, and reduce growth rate of plants |
| Cu | Low doses of Cu are required for normal functioning of the body. High doses cause irritation to mouth, eyes and throat, nausea, vomiting and diarrhoea, liver and kidney damage and may lead to death. | Toxic to aquatic life with toxicity increasing with decreased water hardness |
| Pb | Affects the nervous system, causes anaemia, malnutrition, abdominal pain and colic, damage the brain and kidneys in adults and may cause death, miscarriage and can affect a child's mental and physical growth. | Death, effects reproduction, lowers fertility, accumulates in living tissues and is persistent. |
| Hg | Affects the nervious system, can cause damage to the brain, kidneys, and developing foetus. | Acute and chronic toxicity to aquatic life |
| Ni | Irritation to eyes, nose and throat, stomach aches, and blood and kidney disorders, may cause asthma, potential carcinogen | Acute and chronic toxicity to aquatic life |
| Se | Low doses of Se are required for normal functioning of the body. High doses of Se cause headaches, dizziness, fatigue, irritation and bronchitis. | Plants are tolerant to Se, but can accumulate high concentrations and become toxic to grazing livestock, causing "Blind staggers" and "Alkali disease". |
| Benzene | Carcinogenic, affects blood production, harmful to the immune system and can cause Leukaemia | Toxic to aquatic life, lowers fertility and changes in appearance or behaviour, causes death in plants and roots and damage to the leaves of agricultural crops. |
| Formaldehyde | Irritation to eyes, nose and throat, causes allergies with high exposures leading to death. | Toxic to aquatic life, lowers fertility and changes in appearance or behaviour |
| Dioxins and furans | Causes skin disease (chloracne), liver damage, increases risk of cancer | Toxic to animals and bioaccumulate |
| PAH | Skin irritation and allergy, headaches, nausea, damage the red blood cells, damage the liver and kidneys, probable carcinogens to humans | Toxic to aquatic life and birds, can cause death to agricultural crops |
| Toluene | Light-headedness, dizziness, sleepiness, unconsciousness, brain damage, death | Moderate toxicity to aquatic life, membrane damage to the leaves in plants |
| Xylenes | Irritation to eyes, nose and throat, stomach problems, drowsiness, loss of memory, may damage bone marrow | Toxic to aquatic life, damages agricultural crops |
